# The role of *ALOX5AP*, *LTA4H *and *LTB4R *polymorphisms in determining baseline lung function and COPD susceptibility in UK smokers

**DOI:** 10.1186/1471-2350-12-173

**Published:** 2011-12-29

**Authors:** Asif S Tulah, Stuart G Parker, Miriam F Moffatt, Andrew J Wardlaw, Martin J Connolly, Ian Sayers

**Affiliations:** 1 Division of Therapeutics and Molecular Medicine, Nottingham Respiratory Biomedical Research Unit, University of Nottingham, Queen's Medical Centre, Nottingham, UK; 2 Sheffield Institute for Studies on Ageing, University of Sheffield, Barnsley Hospital NHSFT, Barnsley, UK; 3 National Heart and Lung Institute, Imperial College London, London, UK; 4 Institute for Lung Health and Department of Infection, Immunity and Inflammation, Glenfield Hospital, University of Leicester, Leicester, UK; 5 Freemasons' Department of Geriatric Medicine, University of Auckland, Auckland

## Abstract

**Background:**

We have previously shown evidence that polymorphisms within genes controlling leukotriene B_4 _(LTB_4_) production (*ALOX5AP *and *LTA4H*) are associated with asthma susceptibility in children. Evidence also suggests a potential role of LTB_4 _in COPD disease mechanisms including recruitment of neutrophils to the lung. The aim of the current study was to see if these SNPs and those spanning the receptor genes for LTB_4 _(*LTB4R1 *and *LTB4R2*) influence baseline lung function and COPD susceptibility/severity in smokers.

**Methods:**

Eight *ALOX5AP*, six *LTA4H *and six *LTB4R *single nucleotide polymorphisms (SNPs) were genotyped in a UK Smoking Cohort (n = 992). Association with baseline lung function (FEV_1 _and FEV_1_/FVC ratio) was determined by linear regression. Logistic regression was used to compare smoking controls (n = 176) with spirometry-defined COPD cases (n = 599) and to more severe COPD cases (GOLD stage 3 and 4, n = 389).

**Results:**

No association with *ALOX5AP*, *LTA4H *or *LTB4R *survived correction for multiple testing. However, we showed modest association with *LTA4H *rs1978331C (intron 11) with increased FEV_1 _(p = 0.029) and with increased FEV_1_/FVC ratio (p = 0.020).

**Conclusions:**

These data suggest that polymorphisms spanning *ALOX5AP*, *LTA4H *and the *LTB4R *locus are not major determinants of baseline lung function in smokers, but provide tentative evidence for *LTA4H *rs1978331C (intron 11) in determining baseline FEV_1 _and FEV_1_/FVC ratio in Caucasian Smokers in addition to our previously identified role in asthma susceptibility.

## Background

Chronic obstructive pulmonary disease (COPD) is a complex respiratory disease with genetic and environmental contributors to pathophysiology [1,2]. Evidence suggests the dihydroxy leukotriene, leukotriene B_4 _(LTB_4_), plays a role in this disease as its production is elevated in the airways of COPD subjects [3,4]. The altered inflammatory response of the airways of COPD sufferers is a result of lymphocytes and neutrophils, suggested in part to be the result of cigarette smoke inhalation [5]. Importantly, LTB_4 _has been shown to have chemotactic activity recruiting inflammatory cells to the lung [6,7]. LTB_4 _is implicated in the neutrophillic inflammation of COPD and has been suggested as the major chemotactic agent in more severe forms of this disease [8]. It has been established that the cysteinyl leukotrienes (CysLTs; LTC_4_, LTD_4 _and LTE_4_) play a significant role in bronchoconstriction and airway inflammation in asthma [9] but their role in COPD is less clear.

Studies have suggested that polymorphisms spanning leukotriene pathway genes may be important in determining leukotriene production and susceptibility to allergic disorders, such as asthma [10]. LTB_4 _and the CysLTs are produced from arachidonic acid in a multi-enzyme pathway called the 5-lipoxygenase (5-LO) pathway. Single nucleotide polymorphisms (SNPs) in two 5-LO pathway genes; 5-lipoxygenase activating protein (*ALOX5AP*) and leukotriene A_4 _hydrolase (*LTA4H*) have shown an association with LTB_4 _overproduction from ionomycin-stimulated neutrophils and with myocardial infarction (MI) susceptibility [11,12]. 5-lipoxygenase activating protein (FLAP) with 5-LO is involved in the synthesis of LTA_4 _which can be conjugated with glutathione by LTC_4 _synthase to form LTC_4 _and subsequent CysLTs or converted to LTB_4 _by the enzyme LTA_4 _hydrolase (LTA_4_H) [13]. FLAP is involved in the production of all leukotrienes; however LTA_4_H is specifically involved in LTB_4 _production.

A recent study has suggested that LTA_4_H contains an aminopeptidase activity as well as having a role in LTB_4 _production [14]. This aminopeptidase activity cleaves the neutrophil chemoattractant proline-glycine-proline (PGP), a COPD biomarker, responsible for the influx of neutrophils into the lung - contributing to chronic inflammation. Cigarette smoke was found to inhibit this aminopeptidase activity thereby leading to accumulation of PGP and neutrophils [14]. This dual role could have important consequences for the design of therapeutics targeting LTA_4_H.

We have recently reported evidence that SNPs spanning *ALOX5AP *and *LTA4H *are asthma susceptibility markers [15]. SG13S114A, SG13S89A and SG13S41G (*ALOX5AP*) and rs1978331C (*LTA4H*) were associated with asthma and asthma related phenotypes (atopy, FEV_1_, bronchial hyperresponsiveness) in a family based association study using 341 asthma families with two affected siblings [15]. Several haplotype associations were also observed [15]. To date, no study has investigated the role of these SNPs with respect to COPD or baseline lung function in smokers. Smoking is associated with decline in lung function and is a major risk factor for the development of COPD; we therefore investigated the role of *ALOX5AP*, *LTA4H *and *LTB4R *SNPs in smokers.

The aim of the current study was to determine whether polymorphisms spanning *ALOX5AP*, *LTA4H *and the *LTB4R *locus influence baseline lung function (FEV_1 _and FEV_1_/FVC ratio) in smokers and whether they contribute to susceptibility to develop COPD or a more severe form of COPD in smokers. We genotyped twenty SNPs spanning these three loci in a cohort recruited for COPD or smoking history (n = 992 subjects) and completed a series of association analyses.

## Methods

### Subjects and baseline characteristics

Subjects were recruited from five UK centres for smoking history and/or COPD diagnosis (n = 992) [16]. Subjects collected from Nottingham (n = 537) were Caucasian, > 40 years and had > 10 pack-year smoking history. Subjects collected from other UK centres (n = 455) were recruited for physician and spirometry defined COPD, Caucasian, > 40 years, smokers with > 10 pack-year history. The combined subjects (n = 992) recruited for smoking history or COPD diagnosis was stratified into 'healthy' smokers (smoking controls) (n = 176, post-bronchodilator (BD) salbutamol FEV_1 _> 80% predicted and postBD FEV_1_/FVC > 0.7) and COPD cases (n = 599, postBD FEV_1 _< 80% predicted and postBD FEV_1_/FVC ratio < 0.7). Subjects not meeting these criteria (or with missing data) were excluded from the case control analyses (n = 217). To investigate whether SNPs determined severity of COPD in the smokers we compared smoking controls with postBD spirometry, i.e. the GOLD classifications [17]. Ethical approval was obtained from the relevant ethics committees (Nottingham, Sheffield, Manchester, Leicester and Oxford) and informed consent from all subjects was obtained.

### Selection of SNPs and genotyping

**Figure 1:**
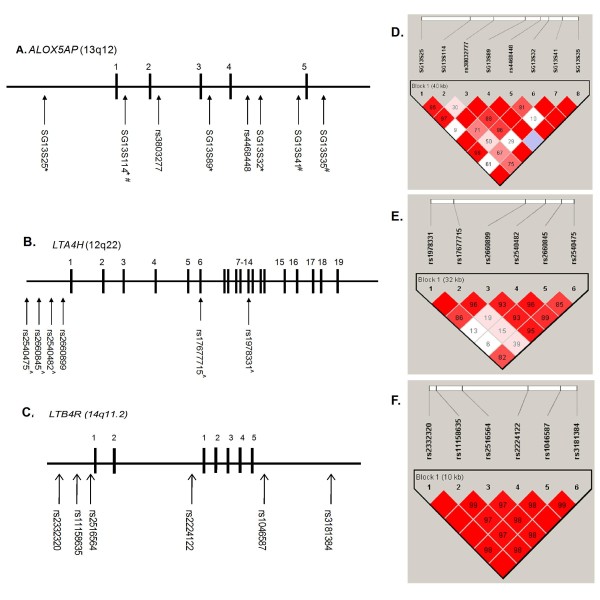
**Location of SNPs genotyped in *ALOX5AP*, *LTA4H *and *LTB4R *in the smokers and linkage disequilibrium (LD) plot**. The LD plot shows the LD displayed as D'/LOD in Haploview software. Numerical values shown correspond to D'. A-C. represents the physical location of the SNPs genotyped in *ALOX5AP*, *LTA4H *and *LTB4R*, respectively. Black boxes represent exons and the spaces between represent introns. D. E. and F. represents the LD plot in the smokers (n = 992) for each loci respectively..* represents SNPs that define the haplotype HapA, #which define HapB and ^ which define the haplotype (HapK) previously associated with MI [11,12].

Twenty SNPs were genotyped across *ALOX5AP *(eight), *LTA4H *(six) and *LTB4R *(six) (Figure 1A-C). SNPs spanning *ALOX5AP *and *LTA4H *have previously been shown to tag haplotypes associated with myocardial infarction and LTB_4 _production [11,12] and with asthma susceptibility in our recent study [15]. Six *LTB4R *SNPs were chosen for their ability to tag linkage disequilibrium (LD) blocks or for inferred function, once the region had been sequenced in Caucasian individuals (22 SNPs validated in 35 Caucasian subjects, data not shown). Genotyping was completed by Kbioscience (Hertfordshire, UK) using KASPar technology. Hardy-Weinberg equilibrium was assessed in all subjects using Haploview software [18].

### Statistical analyses

Linear regression (SPSS V15, SPSS Inc., Chicago, IL) was used to determine the contribution of each SNP to baseline FEV_1 _(litres) or FEV_1_/FVC ratio using the additive model (e.g. AA vs. AC vs. CC) including age, gender, height and smoking pack-years as covariates. The COPD susceptibility analyses were completed using logistic regression in the additive model in two ways. Firstly, the smoking controls (n = 176) vs. all COPD subjects (n = 599) and then the smoking controls (n = 176) vs. GOLD 3 and 4 subjects (n = 389). Both analyses included age, gender and pack-years as covariates (Table 1). Based on the 80 tests completed (analysis of 20 SNPs with 4 outcomes), a conservative Bonferroni correction suggested a p < 10^-4 ^when reporting results as significant. With respect to power (based on lowest and highest minor allele frequency), there was between 77-99% power to detect a 50 ml difference in FEV_1 _and between 58-99% power to detect a 5% difference in FEV_1_/FVC ratio. Analyses of COPD susceptibility were relatively underpowered, with between 28-91% power with an odds ratio of 1.5 and 68-99% power with an odds ratio of 2.0. All analyses considered an error rate of 5%.

**Table 1:** Baseline characteristics of the study populations

	UK Smoking Cohort	Smoking Controls	COPD Cases	GOLD stage 3 and 4	Comparison (smoking controls vs. COPD cases	Comparison (smoking controls vs. GOLD 3/4
Age	63.33 ± 10.29	54.38 ± 9.52	65.96 ± 9.01	67.16 ± 8.56	p < 0.0001	p < 0.0001
Female (%)	43.8	56.3	39.9	38.8	p = 0.037	p = ns
Baseline FEV_1 _% predicted	56.05 ± 28.16	96.03 ± 12.15	40.31 ± 15.63	31.46 ± 8.69	p < 0.0001	p < 0.0001
FEV_1_/FVC Ratio	55.34 ± 17.43	77.30 ± 5.90	46.3 ± 12.5	41.57 ± 11.20	p < 0.0001	p < 0.0001
Post BD FEV_1 _% predicted	59.08 ± 27.14	99.48 ± 11.72	44.65 ± 15.52	35.28 ± 8.90	p = 0.049	p < 0.0001
PostBD FEV_1_/FVC Ratio	55.58 ± 17.71	79.10 ± 5.05	46.2 ± 12.00	41.27 ± 10.46	p < 0.0001	p < 0.0001
Pack Years	43.54 ± 26.05	32.74 ± 20.04	47.61 ± 27.01	47.96 ± 27.85	p < 0.0001	p < 0.0001
GOLD Stage (%):						
• Stage 1	6.9	0.0	0.0	0.0		
• Stage 2	32.6	0.0	34.8	0.0		
• Stage 3	42.4	0.0	45.5	69.9		
• Stage 4	18.2	0.0	19.7	30.1		

Number	992	176	599	389		

FEV_1_, Forced expiratory volume in one second; FVC, forced vital capacity; BD, bronchodilator. Control subjects were defined as having postBD (salbutamol) FEV_1 _> 80% and postBD FEV_1_/FVC > 0.7. Subjects with COPD were defined as having postBD FEV_1 _< 80% and FEV_1_/FVC < 0.7. Individuals who did not meet these criteria were excluded from analyses. Continual variables between groups were compared by Independent T-Test, categorical variables by Pearson chi square.

## Results

### Clinical characteristics and genotyping

Subject characteristics for the smoking controls (n = 176) and COPD subjects (n = 599) and the entire cohort (n = 992) are shown (Table 1). Comparison of the smoking controls (n = 176) and COPD sufferers (n = 599) show differences in baseline lung function (percent predicted FEV_1 _96.03% compared to 40.31%) as anticipated (p < 0.0001). These subjects also showed differences for age, gender and pack-years so these variables were included as covariates in analyses. Genotyping completion rates were > 96% for all twenty SNPs genotyped and did not show deviation from Hardy-Weinberg equilibrium (p > 0.05). Minor allele frequencies for *ALOX5AP *and *LTA4H *SNPs were similar to those observed in our previous study [15].

### Haplotype structure

Figure 1 panels A-C show the location of the SNPs genotyped across *ALOX5AP*, *LTA4H *and the *LTB4R *locus respectively. Panel D shows the linkage disequilibrium (LD) pattern of SNPs genotyped across the *ALOX5AP *gene on chromosome 13q12. Within *ALOX5AP *regions of high LD (measured by D') include between SG13S25 (5'UTR) and SG13S114 (intron 1) and of low LD include between SG13S114 (intron 1) and rs38032777 (intron 2). SNPs defining the region SG13S25 (5'UTR) and SG13S35 (3'UTR) show relatively high LD with all other SNPs. Panel E shows the genotyped SNPs for the *LTA4H *gene on chromosome 12q22. For *LTA4H *there are regions of high LD between rs17677715 (intron 6) and rs2660899 (5'UTR) and rs2540482 (5'UTR) and rs2660845 (5'UTR). The two distal SNPs defining the extended region are not in strong LD with others. Panel C shows the SNPs genotyped across the *LTB4R1 *and *LTB4R2 *genes. There was high LD between all SNPs genotyped in the *LTB4R *locus indicating there was some redundancy in genotyping (Panel F).

### Polymorphisms spanning *ALOX5AP, LTA4H *and *LTB4R *are not associated with baseline FEV_1 _and FEV_1_/FVC in smokers

To assess whether SNPs in *ALOX5AP*, *LTA4H *and *LTB4R *influence baseline lung function in smokers we determined their role in baseline FEV_1 _and FEV_1_/FVC in the entire population (n = 992) using linear regression in the additive model (Additional File 1 Table S1). The FEV_1 _analyses did not identify any significant association with *ALOX5AP*, *LTA4H *or *LTB4R *SNPs. *LTA4H *rs1978331C (intron 11) (p = 0.029, mean FEV_1 _values: TT 1.468 ± 0.039L, TC 1.599 ± 0.034L and CC 1.594 ± 0.057L) and rs2660899 (5'UTR) (p = 0.024; GG 1.580 ± 0.030L, GT 1.504 ± 0.044L and TT 1.192 ± 0.158L) were associated with increased FEV_1_, although this did not meet Bonferroni correction. Analysis with FEV_1_/FVC ratio again did not show any significant association. However, the same *LTA4H *rs1978331C (intron 11) showed p = 0.020 with the same direction of effect, increased FEV_1_/FVC ratio (mean ratio ranging from 53.8% in the major homozygotes to 57.4% in the minor homozygotes).

### Polymorphisms spanning *ALOX5AP*, *LTA4H *and *LTB4R *do not determine COPD susceptibility

To determine whether SNPs spanning *ALOX5AP*, *LTA4H *and *LTB4R *act as determinants of COPD susceptibility in smokers, we completed case-control association analyses comparing the smoking controls (n = 176) with the COPD subjects (n = 599), defined by post-bronchodilator spirometry (Table 2). No significant associations were observed. This analysis was corrected for age, gender and pack-years as these traits were significantly different between the study groups (Table 1).

**Table 2:** *ALOX5AP*, *LTA4H *and *LTB4R *SNPs and COPD susceptibility in smokers.

SNP	Location	Controls (n = 176)				COPD (n = 599)					Additive	
										
		0	1	2	MAF	0	1	2	MAF	p-value	Odds ratio	95%CI
***ALOX5AP***												
SG13S25 (G/A)	5'UTR	138	35	2	0.11	491	100	5	0.09	0.556	0.87	0.55-1.37
SG13S114 (T/A)	Intron 1	92	63	20	0.29	248	282	58	0.34	0.198	1.22	0.90-1.65
rs3803277 (C/A)	Intron 2	54	80	42	0.47	184	303	103	0.43	0.318	0.87	0.65-1.15
SG13S89 (G/A)	Intron 3	162	13	0	0.04	551	45	1	0.04	0.771	0.90	0.43-1.88
rs4468448 (C/T)	Intron 4	100	65	11	0.25	333	226	34	0.25	0.807	1.04	0.76-1.45
SG13S32 (C/A)	Intron 4	42	88	43	0.49	162	302	130	0.47	0.351	0.87	0.66-1.16
SG13S41 (A/G)	Intron 4	149	21	0	0.06	524	62	6	0.06	0.542	0.84	0.47-1.48
SG13S35 (G/A)	3'UTR	146	26	0	0.06	479	97	3	0.09	0.970	1.01	0.60-1.71

***LTA4H***												
rs1978331 (T/C)	Intron 11	66	81	27	0.39	223	257	109	0.40	0.419	0.89	0.68-1.18
rs17677715 (T/C)	Intron 6	115	50	6	0.18	372	188	26	0.20	0.899	1.02	0.72-1.46
rs2660899 (G/T)	5'UTR	130	45	1	0.13	418	158	16	0.16	0.113	1.39	0.93-2.08
rs2540482 (T/C)	5'UTR	107	58	8	0.21	358	195	34	0.23	0.473	1.13	0.81-1.58
rs2660845 (A/G)	5'UTR	92	71	12	0.26	328	224	40	0.26	0.483	0.89	0.65-1.22
rs2540475 (C/T)	5'UTR	108	55	5	0.19	358	186	27	0.21	0.770	0.95	0.67-1.35

***LTB4R2***												
rs2332320 (T/C)	5'UTR	130	34	5	0.13	440	124	13	0.13	0.977	1.01	0.67-1.52
rs11158635 (G/T)	5'UTR	100	57	9	0.23	367	193	26	0.21	0.165	0.78	0.55-1.11
rs2516564 (C/T)	5'UTR	104	59	9	0.22	372	193	25	0.21	0.169	0.79	0.56-1.11

***LTB4R1***												
rs2224122 (C/G)	5'UTR	102	55	9	0.22	363	190	28	0.21	0.232	0.81	0.57-1.15
rs1046587 (G/A)	3'UTR	43	94	36	0.48	155	298	128	0.48	0.566	1.09	0.82-1.45
rs3181384 (C/T)	3'UTR	100	58	10	0.20	363	191	28	0.21	0.111	0.76	0.54-1.07

Logistic regression was used to compare genotype frequencies between smoking controls (n = 176) and total COPD cases (n = 599) using the additive model with the covariates age, gender and pack-years. OR, odds ratio; 95% CI, 95% confidence interval. 0, 1 and 2 represent the number of major homozygote, heterozygote and minor homozygote genotype frequencies.

### Polymorphisms spanning *ALOX5AP*, *LTA4H *and *LTB4R *do not determine susceptibility to develop severe COPD

Subjects (where data available) were stratified according to GOLD classifications using post-bronchodilator lung function (GOLD 1 = 44 individuals, GOLD 2 = 209, GOLD 3 = 273 and GOLD 4 = 117). The phenotypic characteristics of these GOLD stratified COPD patients have been previously reported [16]. We did not observe any significant associations with any of the SNPs tested in the control (n = 176) versus severe COPD (n = 389) analyses. rs3803277 (*ALOX5AP*, intron 2) showed protective association (OR = 0.72, 95% CI = 0.52-0.99, p = 0.045), but this did not survive correction (Table 3).

**Table 3:** *ALOX5AP*, *LTA4H *and *LTB4R *SNPs and severe COPD (defined by GOLD stage 3 and 4) in smokers.

SNP	Location	Controls (n = 176)				GOLD 3 and 4 (n = 389)					Additive	
										
		0	1	2	MAF	0	1	2	MAF	p-value	Odds ratio	95%CI
***ALOX5AP***												
SG13S25 (G/A)	5'UTR	138	35	2	0.11	325	60	2	0.09	0.231	0.72	0.42-1.23
SG13S114 (T/A)	Intron 1	92	63	20	0.29	164	180	36	0.33	0.282	1.21	0.86-1.70
**rs3803277 **(C/A)	Intron 2	54	80	42	0.47	122	203	58	0.42	**0.045**	**0.72**	**0.52-0.99**
SG13S89 (G/A)	Intron 3	162	13	0	0.04	356	32	0	0.04	0.701	0.85	0.36-1.99
rs4468448 (C/T)	Intron 4	100	65	11	0.25	221	146	18	0.24	0.882	0.97	0.67-1.41
SG13S32 (C/A)	Intron 4	42	88	43	0.49	110	199	78	0.46	0.195	0.81	0.58-1.12
SG13S41 (A/G)	Intron 4	149	21	0	0.06	343	38	4	0.06	0.293	0.71	0.37-1.35
SG13S35 (G/A)	3'UTR	146	26	0	0.06	312	61	2	0.09	0.677	0.88	0.49-1.59

***LTA4H***												
rs1978331 (T/C)	Intron 11	66	81	27	0.39	153	165	62	0.38	0.090	0.76	0.56-1.04
rs17677715 (T/C)	Intron 6	115	50	6	0.18	241	124	15	0.21	0.808	1.05	0.71-1.56
rs2660899 (G/T)	5'UTR	130	45	1	0.13	264	109	10	0.17	0.114	1.44	0.92-2.26
rs2540482 (T/C)	5'UTR	107	58	8	0.21	231	129	19	0.22	0.677	1.08	0.74-1.58
rs2660845 (A/G)	5'UTR	92	71	12	0.26	209	154	22	0.26	0.452	0.87	0.61-1.25
rs2540475 (C/T)	5'UTR	108	55	5	0.19	236	118	16	0.20	0.895	0.97	0.65-1.45

***LTB4R2***												
rs2332320 (T/C)	5'UTR	130	34	5	0.13	287	81	8	0.13	0.523	1.17	0.73-1.86
rs11158635 (G/T)	5'UTR	100	57	9	0.23	235	131	16	0.21	0.195	0.77	0.52-1.15
rs2516564 (C/T)	5'UTR	104	59	9	0.22	238	130	15	0.21	0.195	0.77	0.52-1.14

***LTB4R1***												
rs2224122 (C/G)	5'UTR	102	55	9	0.22	228	130	18	0.22	0.365	0.83	0.56-1.23
rs1046587 (G/A)	3'UTR	43	94	36	0.48	95	195	83	0.48	0.857	1.03	0.75-1.42
rs3181384 (C/T)	3'UTR	100	58	10	0.20	230	131	18	0.22	0.183	0.77	0.52-1.13

Logistic regression was used to compare genotype frequencies between smoking controls (n = 176) and GOLD stage 3/4 cases (n = 389) using the additive model with the covariates age, gender and pack-years. OR, odds ratio; 95% CI, 95% confidence interval. 0, 1 and 2 represent the number of major homozygote, heterozygote and minor homozygote genotype frequencies. Black bold indicated (p = ≤ 0.05).

## Discussion

This was the first study to investigate polymorphisms spanning genes involved with LTB_4 _production and activity with lung function and COPD susceptibility in smokers. A UK smoking cohort comprising n = 992 individuals with > 40 years and > 10 pack-years smoking history was used to determine whether SNPs in *ALOX5AP*, *LTA4H *and *LTB4R *influenced baseline lung function and susceptibility to develop COPD in smokers. LTB_4 _has been shown to be important for the inflammation observed in COPD, with this mediator upregulated in COPD subjects [4]. We hypothesised that polymorphisms in these genes may influence susceptibility to develop airway obstruction in smokers that is in part driven by LTB_4_. We have found that polymorphisms spanning *ALOX5AP*, *LTA4H *and the *LTB4R *locus are not associated with lung function or COPD susceptibility in smokers as no SNP survived correction for multiple testing. However, we provide tentative evidence for association between *LTA4H *rs1978331C (intron 11) and lung function measures in these subjects.

We have previously investigated the role of polymorphic variation in the genes of the 5-lipoxygenase pathway *e.g. ALOX5*, *LTC4S*, *CYSLTR1 *in asthma and allergy susceptibility [15,19,20] and as determinants of clinical responses to therapies targeting this pathway [21]. These studies provide accumulating evidence that polymorphic variation in these genes influence disease phenotypes in disorders where leukotrienes play a significant role [10], also confirmed with other non-respiratory diseases *e.g. *MI [11,12]. To date, no study has specifically looked at genetic determinants of leukotriene production/activity in smokers with or without COPD. While no association survived the Bonferroni correction, additive model analyses with rs1978331C (*LTA4H*, intron 11) showed a p = 0.029 with an increase in FEV_1 _and p = 0.020 with FEV_1_/FVC ratio. The mean FEV_1 _and FEV_1_/FVC values for the TC heterozygotes and CC homozygotes were similar, but the presence of the minor C-allele for these genotype groups gave higher trait values when compared to the TT homozygotes, suggesting a dosage effect does not occur. These findings provide tentative evidence suggesting that variants in *LTA4H *may determine lung function in COPD.

We next sought to investigate whether polymorphisms spanning these genes determine susceptibility to develop COPD. Case-control association analyses were completed with 'healthy' control smokers and smokers with physician diagnosed COPD (including spirometry). No significant associations with polymorphisms spanning *ALOX5AP*, *LTA4H *and *LTB4R *were identified. We also completed another case-control analysis involving COPD sufferers at the severe end of the spectrum. GOLD groups 3 and 4 were chosen as this represented the most severe cases based on spirometry. Again no significant associations were observed.

There is an interesting link for *LTA4H *with COPD and asthma; in our group's previous study, we showed preliminary association with rs1978331C (*LTA4H*, intron 11) and asthma susceptibility in 341 families (protection, p = 0.036) [15]. A recent study has shown a similar effect in a different disease; heterozygosity at two *LTA4H *SNPs, one rs1978331 (intron 11), is significantly associated with protection from tuberculosis infection, lower mortality amongst patients with severe tuberculosis infection and protection from the development of severe leprosy disease [22]. These two studies show the same protective direction of association and provide further support for a functionally significant role of rs1978331 or (another SNP tagged by this) in determining LTA_4_H expression or activity.

Suggestive association with COPD (p = 0.02 to 0.05) with four *LTA4H *SNPs within the promoter region (these SNPs were not analysed in our current study) was reported by another group [23]. We have not identified any association with *LTA4H *SNPs located in the 5'UTR (rs2540482, rs2660845 and rs2540475) with lung function or COPD susceptibility in smokers. Interestingly, this group also tested different *ALOX5AP *SNPs to our current study and found no association with COPD [23]. These and our own data provide suggestive support for a role of *LTA4H *SNPs in determining baseline lung function in smokers potentially suggesting a role for genetically determined LTB_4 _in COPD (*LTA4H *converts LTA_4 _to LTB_4_). This may be a result of neutrophilic inflammation being important in COPD and severe COPD [24]. While this study did not show significant protective association with *LTA4H *rs1978331C with lung function in smokers, the same direction of effect was observed with asthma susceptibility in our previous study [15] and with the HapK (rs1978331(T/C), rs17677715(T/C), rs2540482(T/C), rs2660845(A/G) and rs2540475(C/T)) haplotype association that conferred a modest risk of myocardial infarction in Icelandic subjects for rs1978331T [12]. As previously mentioned, a protective effect has also been observed with tuberculosis infection [22].

LTA_4_H has a pro-inflammatory role generating LTB_4 _through its epoxide-hydrolase activity (intracellular) and an anti-inflammatory role through its amino-peptidase activity to breakdown PGP, facilitating resolution (extracellular). Cigarette smoke selectively inhibits the ability of LTA_4_H to break-down PGP leading to neutrophil accumulation and contributing to COPD pathogenesis [14]. rs1978331 may affect the levels of transcription of the *LTA4H *gene. Decreased transcription could lead to decreased protein levels of LTA_4_H which may contribute to the protective physiological effect, through reduction in the formation of the inflammatory LTB_4_. However, this mechanism would lead to the accumulation of PGP and so neutrophillic inflammation, counteracting the situation. LTB_4 _and PGP are both neutrophil chemoattractants [25,26]. rs1978331 may alter splicing efficiency of *LTA4H*. rs1978331 is in intron 11 of the gene and exon 10 and 11 of *LTA4H *contains the zinc-binding domain which is required for both the epoxide hydrolase and aminopeptidase activities [27]. The two functional sites are different but overlapping [28]. Altered splicing in this region could affect the ability of LTA_4_H to generate LTB_4 _and/or degrade PGP. Presence of the C-allele may cause splicing events that reduce LTB_4 _formation, but the aminopeptidase activity may remain functional, which could lead to less neutrophil chemotaxis and so less inflammation. Presence of the T-allele may cause splicing events that lead to increased LTB_4 _production. The T-allele in HapK was functionally associated with LTB_4 _overproduction from ionmycin stimulated neutrophils in MI patients [12]. Other factors could complicate this potential mechanism, such as the lung environments in asthma and COPD and the presence/absence of cigarette smoke. This information could have important consequences for the design of any therapeutics inhibiting LTA_4_H. Reducing LTA_4_H activity will reduce LTB_4 _production, but neutrophilic inflammation will persist as PGP will no longer be degraded. A more selective inhibitor strategy would be required to block LTB_4 _production, but leave the aminopeptidase activity intact. This could take advantage of the different substrate specificities of the non-overlapping regions of the 'active site', small molecules which bind to this hydrophobic part of the site can alter the substrate preference of the aminopeptidase activity [28]. Consideration of both LTB_4 _and PGP and consideration of the SNPs spanning *LTA4H *will be required when designing therapeutics.

This is the first study investigating lung function in smokers and genetic variants specific to genes involved with LTB_4 _production and activity. Overall we have identified that polymorphisms spanning *ALOX5AP*, *LTA4H *and *LTB4R *are not major determinants of lung function in smokers. However, these data highlight the potential importance of *LTA4H *polymorphisms in particular rs1978331C (*LTA4H*, intron 11). Although the rs1978331 association did not survive correction for multiple testing, the previous associations with; asthma/lung function [15], MI [12] and TB [22] suggest it may be a true association of modest effect size and this SNPs does influence LTA4H expression and/or activity. While no association survived the Bonferroni correction, additive model analyses with rs1978331C (*LTA4H*, intron 11) showed a p = 0.029 with an increase in FEV_1 _and p = 0.020 with FEV_1_/FVC ratio. The mean FEV_1 _and FEV_1_/FVC values for the TC heterozygotes and CC homozygotes were similar, but the presence of the minor C-allele for these genotype groups gave higher trait values when compared to the TT homozygotes, suggesting a dosage effect does not occur. For rs1978331 TT versus TC genotype groups there was a 131 ml difference in FEV_1 _and for TT versus CC a 126 ml difference in FEV_1 _was observed. The level of FEV_1 _at a given time depends on 1) the maximum lung function obtained during development, and 2) the rate of decline of lung function with age. Lung function reaches a maximum by age 25-35 years [29]. In smokers the rate of decline in FEV_1 _is accelerated and has been calculated to be ~38.2 ml/year in males and 23.9 ml/year in females [29] therefore the differences observed between LTA4H rs1978331 genotypes can be considered clinically relevant and equate to > 3 years decline in FEV_1_. These findings therefore provide tentative evidence suggesting that variants in *LTA4H *may determine clinically relevant lung function levels in smokers.

It is important to acknowledge the limitations of our study. Other SNPs spanning these large genes could be important. There may also be another functional variant in linkage disequilibrium with rs1978331. We cannot exclude the contribution of polymorphisms spanning other 5-LO pathway genes *e.g. ALOX5*, although existing data did not support their inclusion [20,30,31]. The magnitude of effect of SNPs are modest but in line with the predicted relative risk attributed to a single SNP in a single gene in complex disorders. Finally, the number of individuals used in this study was modest and we have not completed extensive replication in multiple cohorts and so caution is required in the interpretation of our novel findings. To our knowledge these SNPs did not show association with lung function and/or COPD in recent GWAS studies. We have also completed a comprehensive look up of genes previously associated with lung function including *LTA4H *and *ALOX5AP *in 20,288 individuals from the general population (the SpiroMeta consortium) and did not identify these genes as containing major determinants of lung function in this large general population cohort [32].

## Conclusions

In conclusion, these data did not confirm the hypothesis that polymorphisms in genes specific to LTB_4 _production and activity are major determinants of baseline lung function in smokers and do not determine susceptibility to develop COPD. However, rs1978331 (*LTA4H*, intron 11) may have a modest effect on lung function parameters in smokers. Heterozygosity of this polymorphism has previously been correlated with LTB_4 _production, asthma and TB. These findings may be important when considering potential approaches to target *LTA4H *in COPD.

## List of Abbreviations

5-LO: (pathway) 5-lipoxygenase (pathway); 95% CI: 95% confidence interval; ALOX5: 5-lipoxygenase; ALOX5AP: 5-lipoxygenase activating protein; COPD:Chronic obstructive pulmonary disease; CysLT: Cysteinyl leukotriene; CYSLTR1: Cysteinyl leukotriene receptor 1; FEV_1_: Forced expiratory volume in one second: FEV_1_/FVC: Ratio of FEV_1 _to FVC; FLAP: 5-lipoxygenase activating protein; FVC: Forced vital capacity; GOLD: Global Initiative for Obstructive Lung Diseases; LD: Linkage disequilibrium; LTA_4_, B_4_, C_4_, D_4_, E_4_: Leukotriene A_4_, B_4_, C_4_, D_4_, E_4_; LTA4H: Leukotriene A_4 _hydrolase; LTB4R1/2: Leukotriene B_4 _receptor 1/2; LTC4S: Leukotriene C_4 _synthase; MAF: Minor allele frequency; MI: Myocardial infarction; OR: Odds ratio; PGP Proline-glycine-proline; PostBD: Post bronchodilator; SNP: Single nucleotide polymorphism; TB: Tuberculosis; UK: United Kingdom; UTR: Untranslated region.

## Competing interests

The authors declare that they have no competing interests.

## Authors' contributions

IS and AST designed the study and drafted the manuscript. AST completed the statistical analyses. SGP, MFM, AJW and MJC recruited and clinically characterised subjects. All authors contributed to the final version of the manuscript.

## Pre-publication history

The pre-publication history for this paper can be accessed here:

http://www.biomedcentral.com/1471-2350/12/173/prepub

## Supplementary Material

Additional file 1 **Baseline lung function (FEV_1 _and FEV_1_/FVC ratio) and *ALOX5AP*, *LTA4H *and *LTB4R *SNPs in the smokers (n = 992)**. This table shows the results of the association analysis between leukotriene pathway SNPs and baseline FEV_1 _and FEV_1_/FVC using the additive model. Covariates included in the model were age, gender, height and pack years. Associations with p < 0.05 are shown in bold black. Click here for file
