# Supplementary material for: The role of ALOX5AP, LTA4H and LTB4R polymorphisms in determining baseline lung function and COPD susceptibility in UK smokers
Source: BMC Med Genet. 2011 Dec 29;12:173. doi: 10.1186/1471-2350-12-173 (PMC3267686; doi:10.1186/1471-2350-12-173)
Supplement: Additional file 1 — Baseline lung function (FEV1 and FEV1/FVC ratio) and ALOX5AP, LTA4H and LTB4R SNPs in the smokers (n = 992). This table shows the results of the association analysis between leukotriene pathway SNPs and baseline FEV1 and FEV1/FVC using the additive model. Covariates included in the model were age, gender, height and pack years. Associations with p < 0.05 are shown in bold black. [file 1471-2350-12-173-S1.DOC]

# Additional Files

# The role of *ALOX5AP*, *LTA4H* and *LTB4R* polymorphisms in determining baseline lung function and COPD susceptibility in UK smokers

Asif S. Tulah^1^, Stuart G. Parker^2^, Miriam F. Moffatt^3^, Andrew J. Wardlaw^4^, Martin J. Connolly^5^ and Ian Sayers ^1§^

^1^Division of Therapeutics and Molecular Medicine, Nottingham Respiratory Biomedical Research Unit, University of Nottingham, Queen’s Medical Centre, Nottingham, United Kingdom

^2^Sheffield Institute for Studies on Ageing, University of Sheffield, Barnsley Hospital NHSFT, Barnsley, United Kingdom

^3^National Heart and Lung Institute, Imperial College London, London, United Kingdom

^4^Institute for Lung Health and Department of Infection, Immunity and Inflammation, Glenfield Hospital, University of Leicester, Leicester, United Kingdom

^5^Freemasons’ Department of Geriatric Medicine, University of Auckland, Auckland, New Zealand

# Additional files

**Additional File 1, Table S1 - Baseline lung function (FEV_1_ and FEV_1_/FVC ratio) and *ALOX5AP*, *LTA4H* and *LTB4R* SNPs in the smokers (n=992).**

| **SNP** | **Location** |  |  |  |  |  | **FEV_1_** |  |  | **FEV_1_/FVC Ratio** |  |
| --- | --- | --- | --- | --- | --- | --- | --- | --- | --- | --- | --- |
|  |  | **0** | **1** | **2** | **MAF** | **p-value** | **group** | **Value (l, SE)** | **p-value** | **group** | **Value (%, SE)** |
| ***ALOX5AP*** |  |  |  |  |  |  |  |  |  |  |  |
| SG13S25 (G/A) | 5’UTR | 802 | 176 | 9 | 0.11 | 0.595 | - | - | 0.205 | - | - |
| SG13S114 (T/A) | Intron 1 | 423 | 452 | 102 | 0.34 | 0.502 | - | - | 0.518 | - | - |
| rs3803277 (C/A) | Intron 2 | 304 | 483 | 191 | 0.46 | 0.113 | - | - | 0.350 | - | - |
| SG13S89 (G/A) | Intron 3 | 912 | 70 | 3 | 0.04 | 0.983 | - | - | 0.433 | - | - |
| rs4468448 (C/T) | Intron 4 | 556 | 369 | 58 | 0.25 | 0.490 | - | - | 0.790 | - | - |
| SG13S32 (C/A) | Intron 4 | 266 | 490 | 227 | 0.49 | 0.131 | - | - | 0.209 | - | - |
| SG13S41 (A/G) | Intron 4 | 860 | 107 | 8 | 0.06 | 0.496 | - | - | 0.559 | - | - |
| SG13S35 (G/A) | 3’UTR | 809 | 150 | 4 | 0.08 | 0.923 | - | - | 0.891 | - | - |
| ***LTA4H*** |  |  |  |  |  |  |  |  |  |  |  |
| **rs1978331** (T/C) | Intron 11 | 347 | 459 | 166 | 0.40 | **0.029** | 0  1  2 | 1.468±0.039  1.599±0.034  1.594±0.057 | **0.020** | 0  1  2 | 53.8±0.90  56.7±0.80  57.4±1.30 |
| rs17677715 (T/C) | Intron 6 | 608 | 320 | 41 | 0.21 | 0.844 | - | - | 0.909 | - | - |
| **rs2660899** (G/T) | 5’UTR | 700 | 262 | 22 | 0.16 | **0.024** | 0  1  2 | 1.580±0.030  1.504±0.044  1.192±0.158 | 0.051 | - | - |
| rs2540482 (T/C) | 5’UTR | 579 | 341 | 56 | 0.24 | 0.882 | - | - | 0.278 | - | - |
| rs2660845 (A/G) | 5’UTR | 530 | 380 | 70 | 0.27 | 0.607 | - | - | 0.383 | - | - |
| rs2540475 (C/T) | 5’UTR | 586 | 325 | 37 | 0.21 | 0.895 | - | - | 0.811 | - | - |
| ***LTB4R2*** |  |  |  |  |  |  |  |  |  |  |  |
| rs2332320 (T/C) | 5’UTR | 734 | 198 | 25 | 0.13 | 0.305 | - | - | 0.061 | - | - |
| rs11158635 (G/T) | 5’UTR | 597 | 314 | 56 | 0.22 | 0.983 | - | - | 0.903 | - | - |
| rs2516564 (C/T) | 5’UTR | 607 | 317 | 55 | 0.21 | 0.942 | - | - | 0.897 | - | - |
| ***LTB4R1*** |  |  |  |  |  |  |  |  |  |  |  |
| rs2224122 (C/G) | 5’UTR | 594 | 305 | 55 | 0.22 | 0.877 | - | - | 0.900 | - | - |
| rs1046587 (G/A) | 3’UTR | 265 | 481 | 217 | 0.48 | 0.562 | - | - | 0.318 | - | - |
| rs3181384 (C/T) | 3’UTR | 593 | 314 | 58 | 0.22 | 0.989 | - | - | 0.772 | - | - |

Regression analysis was used to investigate the association between leukotriene pathway SNPs and baseline FEV_1_ and FEV_1_/FVC using the additive model. Covariates included in the model were age, gender, height and pack years. Associations with a p<0.05 in bold black.
